# Future opportunities and trends for e-infrastructures and life sciences: going beyond the grid to enable life science data analysis

**DOI:** 10.3389/fgene.2015.00197

**Published:** 2015-06-23

**Authors:** Afonso M. S. Duarte, Fotis E. Psomopoulos, Christophe Blanchet, Alexandre M. J. J. Bonvin, Manuel Corpas, Alain Franc, Rafael C. Jimenez, Jesus M. de Lucas, Tommi Nyrönen, Gergely Sipos, Stephanie B. Suhr

**Affiliations:** ^1^Instituto de Tecnologia Química e Biológica António Xavier, Universidade Nova de Lisboa, OeirasPortugal; ^2^Department of Electrical and Computer Engineering, Aristotle University of Thessaloniki, ThessalonikiGreece; ^3^Center for Research and Technology Hellas, ThessalonikiGreece; ^4^CNRS, UMS 3601 – Institut Français de Bioinformatique, IFB-core, Gif-sur-YvetteFrance; ^5^Bijvoet Center for Biomolecular Research, Faculty of Science, Utrecht University, UtrechtNetherlands; ^6^The Genome Analysis Centre, Norwich Research Park, NorwichUK; ^7^Institut National de Recherche Agronomique, UMR BIOGECO 1202, CestasFrance; ^8^ELIXIR Hub, Wellcome Trust Genome Campus, CambridgeUK; ^9^Instituto de Física de Cantabria, Consejo Superior de Investigaciones Científicas – Universidad de Cantabria, SantanderSpain; ^10^CSC – IT Center for Science Ltd., EspooFinland; ^11^EGI.eu, AmsterdamNetherlands; ^12^European Molecular Biology Laboratory – European Bioinformatics Institute, Wellcome Trust Genome Campus, CambridgeUK

**Keywords:** Grid computing, Cloud computing, life sciences, Big Data, e-infrastructures

## Abstract

With the increasingly rapid growth of data in life sciences we are witnessing a major transition in the way research is conducted, from hypothesis-driven studies to data-driven simulations of whole systems. Such approaches necessitate the use of large-scale computational resources and e-infrastructures, such as the European Grid Infrastructure (EGI). EGI, one of key the enablers of the digital European Research Area, is a federation of resource providers set up to deliver sustainable, integrated and secure computing services to European researchers and their international partners. Here we aim to provide the state of the art of Grid/Cloud computing in EU research as viewed from within the field of life sciences, focusing on key infrastructures and projects within the life sciences community. Rather than focusing purely on the technical aspects underlying the currently provided solutions, we outline the design aspects and key characteristics that can be identified across major research approaches. Overall, we aim to provide significant insights into the road ahead by establishing ever-strengthening connections between EGI as a whole and the life sciences community.

## Introduction

Life sciences have become a data-rich industry and with that, new issues emerge challenging the established ways of doing research. In the new era of Big Data, toward which life sciences are rapidly transitioning, data algorithms and knowledge are becoming increasingly available for all (Costa, 2014; Verheggen et al., 2014; Calabrese and Cannataro, 2015; Griebel et al., 2015). Ever since 2007, when sequencers began giving copious amounts of data, life sciences have been steadily moving toward the analysis of massive data sets (Stein, 2010), by establishing new integrative infrastructures and proposing radical new ways of doing research (White, 2014). European e-infrastructures, and particularly the European Grid Infrastructure (EGI^1^) can play a key role in this transition and shape future research infrastructures to drive life sciences research forward.

A good example of Big Data complexity is the information resources that can be combined to elucidate how bio-molecules interact with each other. As with all complex problems, multidisciplinary approaches are the basis for the discovery of such connections, which can lead for example to the development of new drugs. Structural biology and bioinformatics are key tools providing insight into interactions at the molecular level. However, the rapid expansion of available data on structure, chemistry and dynamics of biomolecules poses new computational challenges. e-infrastructures can facilitate access to tools and applications and provide the required resources to run complex data analyses. Therefore synergies between life sciences and ICT^2^ researchers are fundamental in moving modern research forward.

**FIGURE 1:**
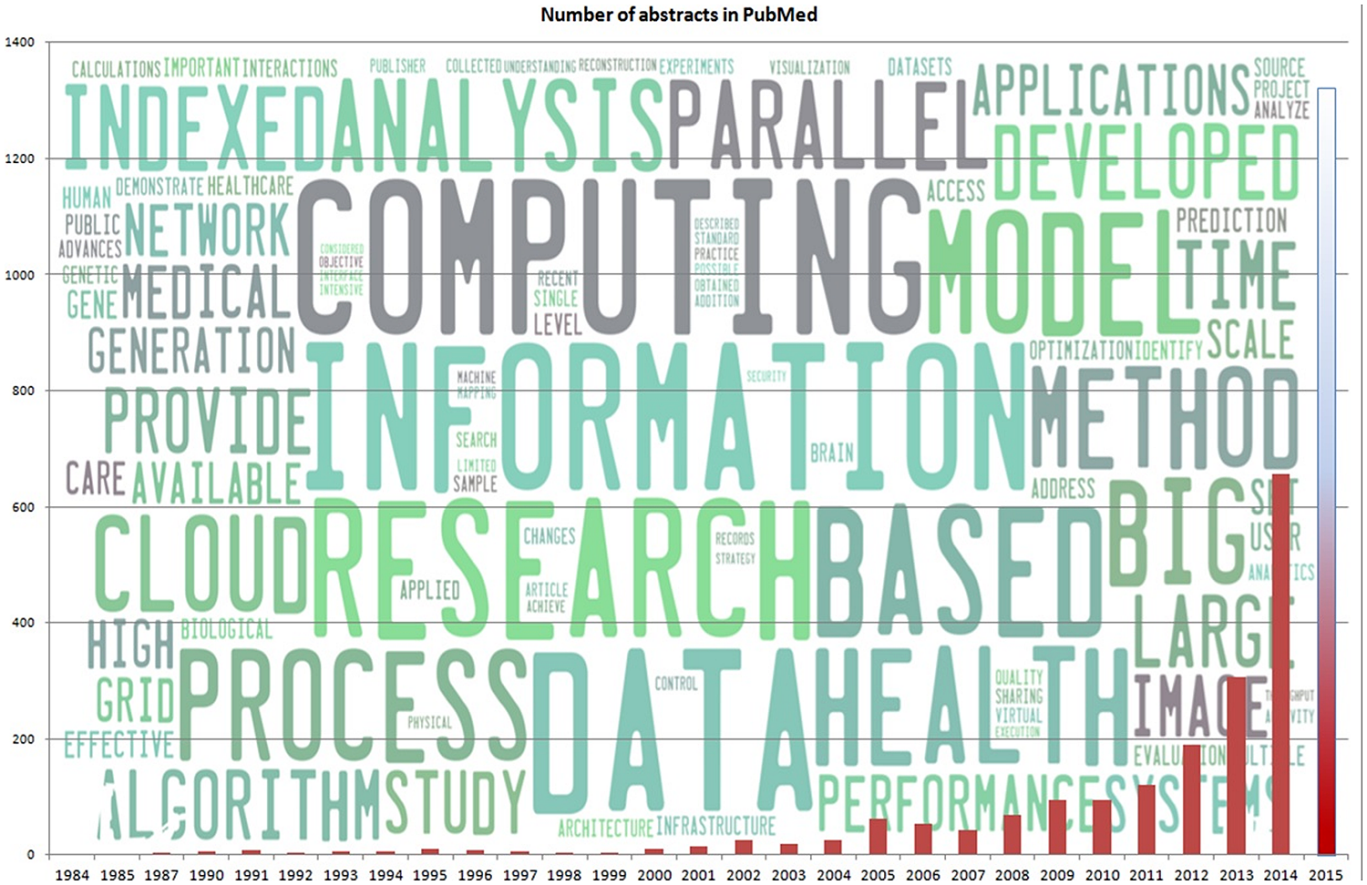
**Number of articles in PubMed related to selected Life Science fields and Big Data technologies (using a low, inclusive threshold).** We searched the PubMed database to reconstruct the story so far. Until April 2015, there are 2125 articles combining “Big Data,” “Grid Computing,” “Cloud computing,” “Parallel Computing,” and/or “Distributed Computing,” following a yearly distribution shown in the figure. It should be noted that the bar corresponding to year 2015 corresponds to the expected number of manuscripts based on linear regression. The actual number of abstracts until April 2015 is 278, which roughly corresponds to half the number of abstracts published in the previous year. It is evident that there is a significant increase in publications, we an ever increasing rate. The word-cloud in the background of the figure has been produced from the retrieved abstracts.

Recently, the emergence of new tools driven by production e-infrastructures such as EGI have given way to an explosion in the number of publications, ranging from large-scale simulations to disease-oriented structural models in proteomics (**Figure 1**). Although these results are far from new, they do point out a critical change: research has shifted radically from hypothesis-driven studies to data-driven simulations of whole systems. At this point it is worth mentioning that the use of large-scale computer resources (e.g., local clusters, super-computers, EU e-infrastructures) is often mislabeled in literature; many results are obtained in local clusters and supercomputers as opposed through distributed collaboration across e-infrastructures. On the other hand, it is becoming quite evident that e-infrastructures combine ICT infrastructure with distributed collaboration aspect. This is a key aspect from which life sciences could hugely benefit from.

European Grid Infrastructure hosted a dedicated workshop and networking session at the EGI Community forum in May 2014 in Helsinki, Finland to facilitate interactions between life sciences and ICT communities. We present here a short overview of the outcomes of this workshop, which include concrete collaborations and sustainable synergies between the two communities, ushering life sciences in the Big Data era with the joint effort of ICT.

## EGI and Life Sciences: Federation Services, Federated Services by EGI for Life Sciences

**FIGURE 2:**
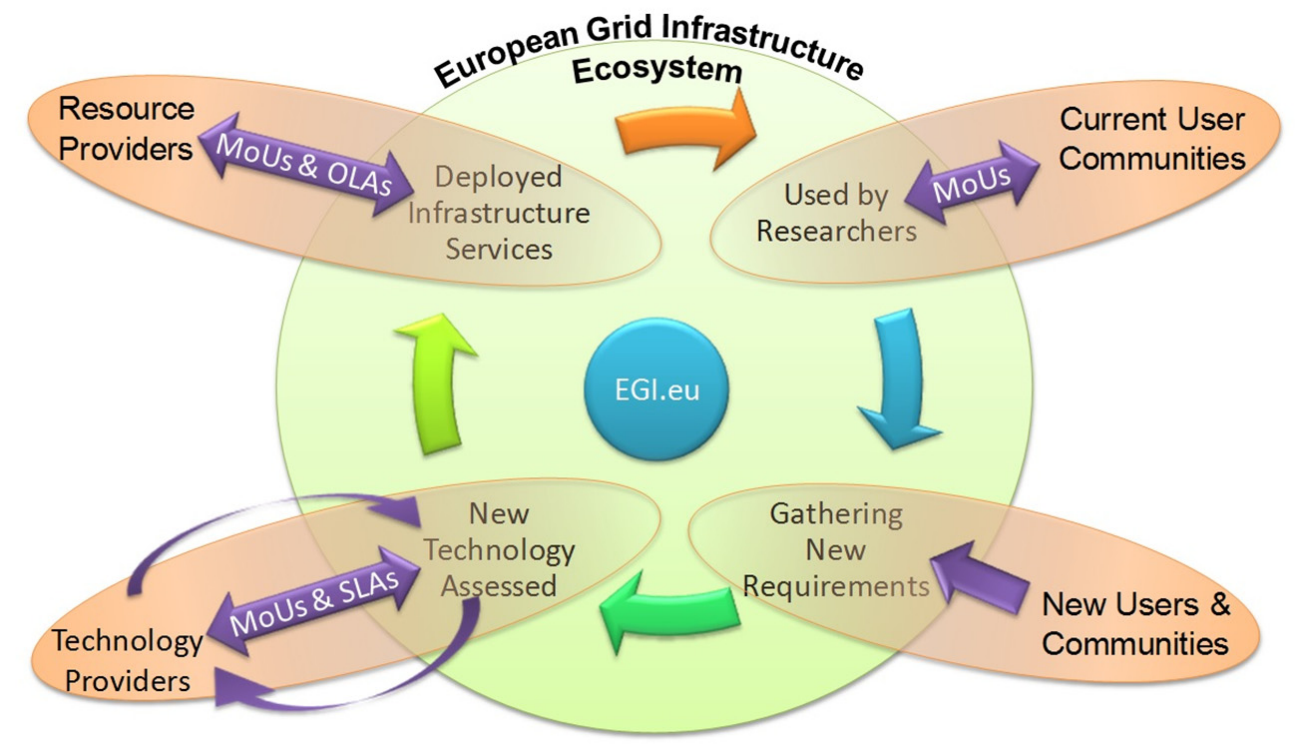
**European Grid Infrastructure (EGI) virtuous service cycle**. The relationship between the key groups (Technology providers, User communities, Resource providers) with the National Grid Initiatives (NGIs), European Intergovernmental Research Organizations (EIROs), and projects (represented through EGI.eu) is presented in a virtuous service cycle. (MoUs, Memorandum of Understanding; OLAs, Operational-level agreements; SLA, Service Level Agreement).

The EGI is the result of pioneering work that has, over the last decade, built a collaborative production infrastructure of uniform services through the federation of national resource providers that supports multi-disciplinary science across Europe and around the world. An ecosystem of national and European funding agencies, research communities, technology providers, technology integrators, resource providers, operations centres, over 350 resource centres, coordinating bodies and other functions has now emerged to serve over 21,000 researchers in their intensive data analysis across over 15 research disciplines, carried out by over 1.4 million computing jobs a day. The evolution of the current EGI services, as well as the introduction of new services is driven by the needs coming from the researchers and infrastructure providers within EGI and the organizations they collaborate with internationally. This process is driven by a virtuous cycle (**Figure 2**). The process includes: the prioritization of requirements, and consequent fulfillment of these requirements by external technology providers, the assessment of the new technology releases (to ensure they meet the original requirements), and the deployment of new technology into the production infrastructure. EGI Federated cloud provides a variety of different custom Virtual Machines that can be customized for the different needs of the end user.

European Grid Infrastructure currently supports an extensive list of services available for life sciences and has been working together with the community to implement further support. EGI is a federation of over 340 resource centres, set up to provide computing and data services and resources to European researchers and their international collaborators. EGI is coordinated by EGI.eu, a non-profit foundation created to manage the infrastructure on behalf of its participants: National Grid Initiatives (NGIs) and European Intergovernmental Research Organizations (EIROs). EGI supports research collaborations of all sizes: from the large teams behind the Large Hadron Collider at CERN and Research Infrastructures on the ESFRI^3^ roadmap, to the individuals and small research groups that equally contribute to innovation in Europe. The EGI Federated Cloud, the latest infrastructure and technological offering of EGI is a prime example of a flexible environment to support both discipline and use case specific Big Data services.

The EGI Federated Cloud is already deployed on nearly 20 academic institutes across Europe who together offer 6000 CPU cores and 300 TB storage for researchers in academia and industry. It currently supports 26 scientific communities and 50 use cases from different scientific disciplines. The technologies that enable the cloud federation are developed and maintained by the EGI community, and are based on open standards. These technologies are available for institutes and communities who wish to setup their own federated cloud infrastructures interfacing the EGI technology with their Cloud Management Frameworks, such as OpenStack or OpenNebula. The EGI Federated Cloud currently supports 26 scientific communities and 50 use cases coming from different scientific disciplines: bioinformatics, physics, earth sciences, basic medicine, arts, language and architecture, mathematics, computer sciences, etc. Furthermore, between 2015 and 2017 several research infrastructures from the ESFRI roadmap (BBMRI, EPOS, ELIXIR, DARIAH, INSTRUCT, LifeWatch, and EISCAT-3D) will define and implement community-specific capabilities on this platform in the recently started H2020 EGI-Engage project. Besides the Federated Cloud infrastructure EGI resource centres also make available approximately 500,000 CPU cores, 200 PB disk, and 300 PB tape storage capacity through various grid middleware solutions. This capacity is clustered into nearly 200 resource pools and is mostly to discipline-specific and regional scientific experiments and projects. Some of the resource pools are available for individual researchers and small research teams, often referred to as the ‘long tail of science.’

## Life Science Community and Its Relation with Grid and HPC

### The Need for HPC in Biodiversity Studies

Diversity is an iconic characteristic of living world (Mayr, 1985). Rooted in Natural History, there is currently evidence of an increasing modeling trend toward genetic diversity and molecular evolution, connecting with system biology through diversity of proteins or metabolites and their interactions. Organizing biodiversity data is a challenge as life is not a random assembly of molecules (Gaston, 2000). New sequencing technologies have deeply revolutionized the approach to diversity, as diversity of genomes is an imprint of diversity of organisms. Molecular data can now be produced with high throughput (currently millions of sequences in one experiment). Many challenges exist for organizing biodiversity data, and here are a few. There are efficient algorithms for most of the tasks in biodiversity: e.g., multiple alignment, phylogenetic inference, clustering, unsupervised or supervised, machine learning for pattern recognition, etc. Each reaches a limit, either in time or memory, for data produced by NGS^4^. EGI provides a unique infrastructure for this challenge, as several of these tasks can be distributed. A key example of such case is the aggregative nested clustering [ASC (Hartigan, 1975, Jain et al., 1999)] on large data sets (between 10^5^ and 10^6^ specimen), which act as a surrogate for molecular phylogenies, reconciling Natural History knowledge, and molecular evolution.

## Life Science e-Infrastructures within EGI: Working Synergies

### The WeNMR Case

The major aim of the WeNMR project^5^ is it to serve the structural biology community in life sciences with innovative and user-friendly e-Science solutions. Structural biology is concerned with the determination of the three-dimensional structures of bio-macromolecules and their complexes. The field contributes to society by supporting a wide range of applications that include drug design, crop improvement, and engineering of enzymes of industrial significance. Its present challenges include the integration of data from various methods, gaining access to sufficient computational resources, developing off-the-shelf solutions for non-experts and dealing with large data sets.

As an EU-funded project, WeNMR (Wassenaar et al., 2012) aimed and succeeded in optimizing and extending the use of Nuclear Magnetic Resonance (NMR) and Small-Angle X-ray Scattering (SAXS) to determine the structure and properties of proteins and other medically important molecules. For this, a Virtual Research Community (VRC) scientific gateway was established^5^; offering training material, a support center, standard workflows, services through easy-to-use web interfaces, and a single-sign-on mechanism. WeNMR brings together a diverse group of stakeholders and has tight connections with various European Research Infrastructure projects^6^ (e.g., BioNMR) After more than 3 years of operations, the WeNMR VRC has grown to over 1700 users and its Virtual Organization^7^ (VO) over 650 users from 42 countries worldwide (36% outside Europe). The resources offered by WeNMR are widely used by the community, which translates into a sustained and increasing number of computational tasks (>2.5 million per year) being sent mainly to Grid resources. It is mostly due to the user-friendly access to software solutions and the computational resources of the grid that users are attracted, together with the excellent support and training offered.

### The LifeWatch Scenario

LifeWatch^8^ is an ESFRI initiative in biodiversity and ecosystem research, providing an exploratory research environment that allows scientists to find data, combine resources, compose workflows, run analyses, develop models, and visualize predictions. As an ESFRI, LifeWatch is benefiting from the framework provided by EGI to set up services satisfying the researcher’s needs. User-friendly services are required to combine data, using established standards and unique identifiers. Flexibility to deploy these services is offered through Grid- or Cloud-based infrastructure. The final ambition is to build a realistic model for the Biosphere. The first example presented in the talk, the project on Monitoring Cyanobacterial Blooms developed in the area of water quality in collaboration with an SME, Ecohydros, for the ROEM+^9^ Life project, is a very good example of the interlink required among different techniques and measurements, and the difficulties to build a realistic model, that requires significant computational resources.

LifeWatch will benefit from a close collaboration with EGI. In particular, the core-ICT team in LifeWatch is deploying its Grid/Cloud enabled infrastructure throughout 2014, and will integrate it in the EGI framework. However, LifeWatch also requires services that are under further development, starting with a common identity federation for researchers, educators and students, the support for unique identity of digital objects, easy deployment of medium and large DBMS instances, integration of GIS systems, or a parallel data mining framework accepting Python and/or R scripts. In Europe, ESFRI initiatives are conceived to address and coordinate large and global challenges in science, going beyond a single research institution, or even a national effort. The framework provided by EGI appears ideal to support the computational needs required by the complex services that LifeWatch is implementing, and a close collaboration over the following years is expected.

## Bridging the Gap: the BioMedBridges Initiative

It is clear that life science research infrastructures and e-infrastructures must work closely together to be able to address the new challenges connected to the dramatic increase of data that is being generated. However, looking more closely, it is not trivial to determine specific requirements and how they may be addressed. This arises from the fact that so far the involved actors do not share the same technical language. To some extent, there is great variation concerning data and possible approaches even among different life science disciplines (e.g., genomic and imaging data). BioMedBridges^10^ is addressing this communication gap with a series of targeted workshops bringing e-infrastructure representatives and personnel from the emerging life science research infrastructures on the ESFRI roadmap together.

These workshops focussed on challenges and possibilities around the “data deluge” faced in life sciences in the near future including storage, transfer, and analysis^11^, and developing suitable data strategies for research infrastructures^12^. Going forward, each of the many different communities within the life science domain must increasingly consider what data is worth storing and accessing, and subsequently define their needs.

## ELIXIR

European countries, companies and funding bodies invest heavily in biological research, seeking solutions to the many serious challenges facing society today^13^. This research produces vast amounts of data at a continuously increasing rate; it has been estimated that by 2020 these data will be generated at up to 1 million times the current rate. At the core of ELIXIR’s strategy is the recognition that large-scale data management in the life sciences is not limited to a few sites. A European data infrastructure must be able to cope with the aggregation, annotation, and integration of data from thousands of laboratories as well as scaling these data-services to millions of users worldwide. ELIXIR brings together EMBL-EBI and national bioinformatics expertise throughout Europe to create a coordinated infrastructure whose contributors share responsibility for biological data delivery, sustainability and management.

ELIXIR has progressed through its preparatory and interim phases, which were focussed on developing sustainable governance and funding models and coordinating the efforts from more than 120 institutions involved in the provision of bioinformatics services through the creation of ELIXIR Nodes. In 2015 ELIXIR moves into its Service Deployment Phase, which will see the implementation of the ELIXIR programme^14^ ELIXIR recognizes that collaboration between partners, the community and research infrastructures like EGI is key in order to build effective and coordinated services for users to share and compute biological data in Europe.

### Bringing the Tools to Data - Provide Scientists with Personalized and On-Demand Bioinformatics Services on the Cloud

Thanks to the continuous improvement of experimental technologies, life science researchers face a deluge of data, the exploitation of which requires large computing resources and appropriate software tools. They simultaneously use many of the bioinformatics tools from the arsenal of thousands available within the international community. Usually, they combine their data with public data that are too large to be moved easily. Therefore, the computational infrastructure needs to be tightly connected to public biological databases.

One important aspect of deploying a cloud for life science is to provide virtual machines (appliances) that encapsulate the many complex bioinformatics pipelines and workflows needed to analyze distributed life science data. At the IFB, we developed several bioinformatics services available as cloud appliances. A cloud appliance is a predefined virtual machine that can be run on a remote cloud infrastructure. As their size is usually in the range of gigabytes, it is more efficient to move the appliance to the location of the terabytes of biological data to be analyzed, instead of moving the data to the appliance. However, this approach requires at least few of the computing resources to be available close to the stored data. We have created bioinformatics appliances providing, for example, a user-devoted Galaxy portal, a virtual desktop environment for proteomics analysis or a bioinformatics cluster with a lot of standard tools. Scientists can run their own appliances through a user-adapted web interface. Moreover, to connect our cloud infrastructure to existing public biological databases, we have configured it to automatically link all virtual machines to a local repository with core public databases like UNIPROT or EMBL.

### Delivering ICT Infrastructure for Biomedical Research

As Biomedical science data volumes grow, local computational resources needed/required to satisfy their processing quickly become insufficient. In addition to computing services and technical support, users need significant storage capacities, and access to large reference data to reflect their findings in the context of the current knowledge. The size of the datasets in biomedical science like the human genetic variation 1000 Genomes, The Cancer Genome Atlas (TCGA) and the Finnish sequencing initiative data are hundreds of terabytes to petabytes in size and grow rapidly. Data capacity challenges form a major research bottleneck.

The CSC – IT Center for Science (CSC) Infrastructure provides a Service (IaaS) cloud concept developed in 2011–2013 in collaboration with biomedical research organizations (Nyrönen et al., 2012). The services are part of the construction of the ELIXIR Finland research infrastructure and are included in the national research infrastructure 2014–2020 roadmap.

Life science service providers typically analyze and integrate high-throughput data, visualize data, share analysis sessions and save and share tool workflows. Tool environment must evolve with the state-of-the-art. The FIMM environment is packaged into virtual machine images and, in theory, any third party can run and support the data analysis infrastructure. Adding and removing capacity from cloud is straightforward, since the building of the data analysis tool environment supports virtualisation.

## Perspectives

The current trend for life science ICT infrastructure uptake is to use the most diverse and state-of-the-art computing technologies (Grid, Cloud, and now Mist among others) to access computable storage, memory and compute in each country/resource federation that can provide these services. The computational requirements of most standard workflows continue to rise. A characteristic case is the analysis of NGS data; READemption, a pipeline for the computational evaluation of RNA-Seq data (Förstner et al., 2014), requires from 2/3 VMs in normal use, which can rise up to around 30 VMs at peak performance with more than 20 cores, more than 70 GB of memory, and around 1 TB of storage. Chipster, a user-friendly analysis software for high-throughput data (Kallio et al., 2011), exhibits similar characteristics where each server requires over four virtual CPUs, over 16 GB of RAM and around 500 GB of storage at each site. These requirements can be addressed through the use of ICT infrastructures such as EGI and be made available to the wider scientific community.

It is becoming more than evident that the future of life sciences applications lies with the use of Grid and Cloud computing. EGI offers a set of solutions to accelerate research through a diverse service catalog: from its Federated Cloud solution^15^ to resource allocation (through the e-GRANT portal^16^), EGI provides life science researchers with a flexible and strong computational set of resources to support scientific excellence at an international level. In the near future, EGI will continue to engage and support the experts in life sciences and ICT communities, helping them to collaborate and better understand each other’s goals.

One current solution for the life science community service providers is virtualisation, i.e., Cloud Infrastructure as a Service (IaaS), to virtualise the local data analysis infrastructure. In our experience this leads to a better division of work between specialized parties to support life sciences. Collaboration focused e-infrastructure virtualisation technologies seems thus natural, and it can allow life sciences to spend more time on data features of the services and less on operating the IT infrastructure. For example, scalability of interactive visualizations for sequencing data for thousands of users cannot be achieved without expert IT infrastructure supporting the overall service delivery.

The challenge that e-Infrastructures face today is how to deliver ICT services supporting high-volume life science data analysis. In the way IT infrastructure is currently delivered, adding capacity such as computable storage to support local data operations is a bottleneck. Solving this challenge is a critical part of the infrastructure for Europe’s life science research sector. By consistently building on national strengths and strategies across Europe, a standardized process of performing large scale computations from the local bioinformatics capacities to the EU-level e-infrastructure will establish the way to drive life science forward.

## Conflict of Interest Statement

The authors declare that the research was conducted in the absence of any commercial or financial relationships that could be construed as a potential conflict of interest.

## Footnotes

1 http://www.egi.eu

2 ICT: Information Communication Technologies

3 ESFRIs: European Strategy Forum on Research Infrastructures

4 Next Generation Sequencing

5 http://www.wenmr.eu/

6 http://www.bio-nmr.net/

7 enmr.eu

8 http://www.lifewatch.eu/

9 http://www.roemplus-life.eu/

10 http://www.biomedbridges.eu/

11 REPORT: BioMedBridges workshop on e-Infrastructure support for the life sciences – Preparing for the data delugehttp://dx.doi.org/10.5281/zenodo.13942

12 REPORT: BioMedBridges workshop on e-Infrastructure support for the life sciences – Preparing for the data delugehttp://dx.doi.org/10.5281/zenodo.13942

13 http://elixir-europe.org/

14 https://www.elixir-europe.org/about/elixir-programme-2014-2018

15 http://www.egi.eu/how-to/use_the_federated_Cloud.html

16 http://e-grant.egi.eu/
